# Male-benefit sexually antagonistic genotypes show elevated vulnerability to inbreeding

**DOI:** 10.1186/s12862-017-0981-4

**Published:** 2017-06-12

**Authors:** Karl Grieshop, David Berger, Göran Arnqvist

**Affiliations:** Department of Ecology and Genetics, Animal Ecology, Uppsala University, Norbyvägen 18D, 752 36 Uppsala, Sweden

**Keywords:** Antagonistic pleiotropy, Balancing selection, Fitness, Genetic variation, Inbreeding depression, Intralocus sexual conflict, Mutation load, Sexually antagonistic selection

## Abstract

**Background:**

There is theoretical and empirical evidence for strong sexual selection in males having positive effects on population viability by serving to purify the genome of its mutation load at a low demographic cost. However, there is also theoretical and empirical evidence for negative effects of sexual selection on female fitness, and therefore population viability, known as the gender load. This can take the form of sexually antagonistic (SA) genetic variation where alleles with a selective advantage in males pose a detriment to female fitness, and vice versa. Here, using seed beetles, we shed light on a previously unexplored manifestation of the gender load: the effect of SA genetic variation on tolerance to inbreeding.

**Results:**

We found that genotypes encoding high male, but low female fitness exhibited significantly greater rates of extinction upon enforced inbreeding relative to genotypes encoding high female but low male fitness. Also, genotypes encoding low fitness in both sexes exhibited greater rates of extinction relative to generally high-fitness genotypes (though marginally non-significant), an expected finding attributable to variation in mutation load across genotypes. Despite follow-up investigations aiming to identify the mechanism(s) underlying these findings, it remains unclear whether the gender load and the mutation load have independent consequences for tolerance to inbreeding, or whether these two types of genetic architecture interact epistatically to render male-benefit genetic variation relatively intolerant to inbreeding.

**Conclusions:**

Regardless of the underlying mechanism(s), our results show that male-benefit/female-detriment SA genetic variation poses a previously unseen detriment to population viability due to its elevated vulnerability to inbreeding/homozygosity. This suggests that sexual selection in the context of SA genetic variance for fitness may enhance the gender load on population viability more than previously appreciated, due to selecting for male-benefit SA genetic variation that engenders lineages to extinction upon inbreeding. We note that our results imply that SA alleles that are sexually selected for in males may be underrepresented or even lacking in panels of inbred lines.

**Electronic supplementary material:**

The online version of this article (doi:10.1186/s12862-017-0981-4) contains supplementary material, which is available to authorized users.

## Background

Sexual selection can purge the genome of its mutation load on population viability, and at low demographic costs when via males [1–6]. This requires that deleterious mutations have sexually concordant (SC) effects, affecting both sexes similarly, such that the mutations that are selectively sieved out of a population due to the detriments they pose to male fitness are the same mutations that would harm population viability by causing reductions in female offspring production and/or juvenile survival [6]. If sexual selection is strong enough, this process poses a population-level viability advantage over asexual invaders [6]. In theory, this can counter the two-fold transmission advantage of asexual genomes, offering a population-level adaptive explanation for the prevalence and maintenance of sexual reproduction in eukaryotes [6]. This explanation for the maintenance of sex [6] and some supporting empirical evidence (e.g. [7–11]) has caused sexual selection to become viewed as a phenomenon that, if anything, likely elevates population viability.

However, as SC genetic variation for fitness is eroded by purifying selection, we expect populations to become dominated by sexually antagonistic (SA) genetic variance for fitness [12]. SA genetic variation is characterized by genotypes encoding high fitness in one sex but low fitness in the other [13–18] and is expected to contribute disproportionately to genetic variance for fitness in well-adapted populations [12, 19–21]. Because female offspring production is a crucial aspect of population viability, a paradox emerges: while sexual selection in males may promote population viability by purifying the genome of its SC mutation load [6], the SA genetic variation left behind [12] can pose a severe gender load on the population [15, 22–24]. For example, in a wild-caught population of the seed beetle *Callosobruchus maculatus* dominated by SA genetic variance for fitness [25], we have shown that genotypes exhibiting high male fitness exhibit low potential to contribute positively to population growth rate [26]. Indeed, Whitlock and Agrawal [6] point out that the capacity for strong sexual selection in males to purge a population’s mutation load will depend critically on what proportion of the genetic variance for fitness is SA (as well as the degree of phenotypic sexual conflict), where a strong enough gender load could nullify or even reverse the population-level benefits of sexual selection in males.

Here, using that same population of seed beetle described above, we evaluate the potential for SA genetic variation for fitness to pose population-level detriments via another important component of population viability: tolerance to inbreeding depression. Inbreeding depression occurs due to increasing homozygosity for weakly deleterious mutations throughout the genome, which are (partially) shielded from purging by virtue of being rare and partially recessive [27–30]. Indeed, Jarzebowska and Radwan [9] showed that while sexual selection in males can act to remove large-effect mutations, posing short-term tolerance to inbreeding depression, it may be ineffective at removing much of the small-effect mutations that contribute to a mutation load on population viability. Thus, even populations dominated by SA genetic variance for fitness will exhibit a SC mutation load on population viability, despite their presumed history of purifying sexual selection, and this mutation load is expected to cause inbreeding depression upon increasing homozygosity [28–30]. However, it is currently not clear how this SC mutation load will interact with SA genetic variation upon increasing homozygosity.

Because female offspring production plays an obvious role in buffering the population-level detriments of inbreeding depression, one general prediction is that male-benefit/female-detriment genotypes will contribute greater detriments to populations upon increasing homozygosity relative to female-benefit/male-detriment genotypes. Here, we test this prediction by measuring lineage extinction under enforced inbreeding among SA genotypes. The population of seed beetle used for this experiment exhibits predominantly SA genetic variance for fitness [25, 26] and we have demonstrated strong sexual selection in males against an induced mutation load on female/population offspring production in this population [11]. We therefore expect that this population has experienced a history of strong sexual selection in males that has contributed to purging some of its SC mutation load, and therefore also contributed to generating its predominantly SA genetic variance for fitness.

We subjected 20 replicate lineages from each of 41 isofemale lines (~800 lineages at the start) to single-pair full-sib inbreeding for 10 consecutive generations or until lineage extinction. We predicted that lineages originating from isofemale lines enriched for male-benefit/female-detriment alleles would exhibit greater rates of extinction relative to lineages originating from female-benefit/male-detriment isofemale lines. Further, if lineage extinction results from increased homozygosity, then variation in deleterious recessives should cause lineages from generally low-fitness isofemale lines (i.e. low-male and low-female fitness) to likewise exhibit greater extinction rates relative to lineages from generally high-fitness isofemale lines [28–30]. Our findings support these predictions, demonstrating an additional mechanism by which sexual antagonism can impose a gender load on population viability.

## Methods

### Study organism

*C. maculatus* (Coleoptera: Bruchidae) is a pest of leguminous crops that has colonized most of the tropical and subtropical regions of the world [31]. Males and females are sexually mature upon adult eclosion, and exhibit a polyandrous mating system [32]. The eggs are glued onto the surface of dry beans and hatched larvae bore into the beans, where they complete their life cycle. *C. maculatus* are facultatively aphagous; adults require neither food nor water to reproduce successfully.

### Study population

The population of 41 isofemale lines used in our inbreeding experiment (below) was previously shown to exhibit significant SA genetic variance for fitness under normal environmental conditions [25, 26]. The population was isolated from *Vigna unguiculata* seed pods collected at a small-scale agricultural field close to Lomé, Togo (06°10'N 01°13'E) during October and November, 2010. Seed pods were stripped in the laboratory and beans isolated individually. Virgin males and females hatching out of these beans were paired randomly and each pair founded an isofemale line (*n* = 41), each of which was thus derived from a single maternal and a single paternal genome. The F_1_ offspring from each of these pairs/isofemale lines were allowed to breed with their siblings in order to expand the isofemale lines. The isofemale lines were cultured at a population size of 200–300 adults each generation on 150 ml. of *V. unguiculata* seeds at 29° C, 55% RH and a 12 L:12D light regime until the start of experiments. Thus, inbreeding upon the establishment of the isofemale lines, as well throughout their maintenance until the start of experimentation, was limited to that one original F_1_ generation. Our isofemale lines thus exhibit segregating genetic variation that can be driven to fixation upon inbreeding. We note that no isofemale line went extinct during the establishment phase.

**Fig. 1:**
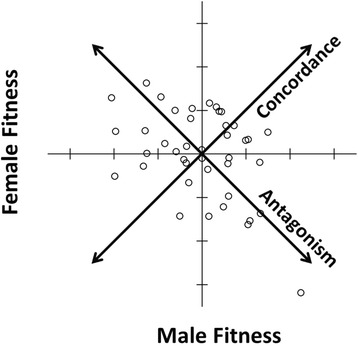
Intersexual genetic correlation for fitness among the 41 isofemale lines used here, demonstrating abundant SA genetic variance (see [25]), as well as adequate representation throughout the distribution of all four variables. Derived axes/variables, antagonism and concordance (see Methods), represent continuums ranging from extreme female-benefit/male-detriment isofemale lines (quadrant 2) to extreme male-benefit/female-detriment isofemale lines (quadrant 4), and from generally low-fitness (i.e. low-male/low-female) isofemale lines (quadrant 3) to generally high-fitness (i.e. high-male/high-female) isofemale lines (quadrant 1), respectively.

Isofemale lines were cultured for 12 generations prior to the sex-specific fitness assays [25] . These assays are described in detail in Berger et al. [25]. Briefly, for males, a single virgin male from a given isofemale line was placed in a petri dish (90 mm ∅) containing *ad libitum V. unguiculata* seeds together with 2 virgin irradiated (sterile) males from a standard reference population (whose sperm function and fertilize eggs, but whose zygotes die due to lethal mutations) and 3 virgin females from the reference population (a 1:1 sex ratio). Thus, the male fitness assays included mating competition and sperm competition. For females, a single virgin female from a given isofemale line was placed in a petri dish (90 mm ∅) containing *ad libitum V. unguiculata* seeds together with 2 virgin males from the reference population, challenging a female’s ability to endure and fend off repeated mating attempts by males. Male and female fitness assays were placed in an incubator with the same abiotic conditions described above until all of the F_1_ offspring emerged. Fitness was defined as the lifetime offspring production in these assays. This population of isofemale lines exhibited a significantly negative intersexual genetic correlation for fitness (*r*_MF_ = −0.51; Fig. 1) [25]. Berger et al. [25], having revealed significant genetic variance for female fitness, were unable to detect significant genetic variance for male fitness due to marked environmental variance in male reproductive success. To address this issue, 10 generations after the original fitness estimates from Berger et al. [25], the 5 top and 5 bottom lines were re-assayed. This revealed a highly significant correlation for male fitness between the old and new estimates (*r* = 0.80, *n* = 10, *P* < 0.001), demonstrating that this population does indeed exhibit significant genetic variance for male fitness.

### Inbreeding experiment

The inbreeding experiment began after the completion of the sex-specific fitness assays (see above [25]). 20 replicate lineages of each of the 41 isofemale lines (with minor exceptions, see Additional file 1: S1) were initiated by a single virgin mating pair placed in a petri dish (90 mm ∅) containing *ad libitum V. unguiculata* seeds, for a starting total of 804 lineages. These lineages were placed in incubators with the same abiotic conditions described above. After these parents died, egg-laden seeds were selected and isolated to harvest virgin offspring. Upon emergence, a random selection of virgin full-sib offspring were placed in a small empty petri dish (30 mm ∅) until copulating pairs formed (typically several formed in < 1 min), and a single pair (while still in copula) was gently transferred to a fresh petri dish (90 mm ∅) containing *ad libitum V. unguiculata* seeds. Thus, a single virgin full-sib mating pair from each lineage was used to parent the next generation. This was repeated for 10 consecutive generations or until the lineage went extinct. Extinctions were never attributable to the absence of copulation, as all copulations were witnessed (but see Additional file 1: S2).

### Causes of extinction

**Fig. 2:**
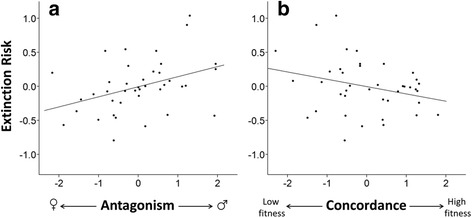
Each isofemale line’s intercept as a random effect in a null Cox regression (“Extinction Risk”) plotted against (**a**) Antagonism and (**b**) Concordance (see Methods and Fig. 1), demonstrating, respectively, that male-benefit/female-detriment and generally low-fitness (i.e. low-male/low-female) isofemale lines were more sensitive to inbreeding relative to their respective opposite extremes. Linear relationships (for visual purposes only) provided by ordinary least squares regression.

Determining the proximate causes of extinction is, for rather obvious reasons, very difficult [33]. Yet, we conducted a follow-up experiment to shed light on the extent to which patterns of extinction could be attributed to variation in female fecundity or male fertility *per se* across the different isofemale lines of origin. After 10 generations of inbreeding, the 3 isofemale lines with the most positive, and 3 with the most negative, score along the first principle component extracted from the bivariate regression of ‘antagonism’ on ‘extinction risk’ (see Fig. 2a and *Statistical analysis*, below) were identified as the 3 most male-benefit/high-extinction isofemale lines and 3 most female-benefit/low-extinction isofemale lines, respectively. We note that all 20 replicate lineages of three of the most male-benefit/high-extinction isofemale lines had gone extinct after 10 generations of inbreeding; thus this follow-up experiment lacks representation from some of the most extremely male-benefit/high-extinction isofemale lines. In these cases, the isofemale lines with the next-most positive score along the principle component describe above were used instead. Two representative extant lineages from each of these extreme 6 (i.e. 3 ‘male-benefit’ and 3 ‘female-benefit’) isofemale lines were randomly selected for use in assays of female fecundity and male fertility—for some extreme isofemale lines, the 1 or 2 extant lineages remaining were the only available option.

Representative lineages selected for this follow-up experiment were expanded to a population size of approximately 200–300 adults that were then kept on 150 ml. of *V. unguiculata* seeds at the same abiotic conditions described above. Parents of these populations were allowed to oviposit for 2 days, at which point egg-laden seeds were isolated to harvest virgin offspring. Virgin male and female focal individuals from representative lineages were mated to virgin individuals from an outbred base population established at the same time, and from the same population, as the isofemale lines (see above). For logistic purposes, the base population was cultured in temporally staggered cohorts. The base population cohort to which focal individuals were mated was recorded. Mated pairs (i.e. focal females crossed to base population males, and focal males crossed to base population females) were placed in a petri dish (90 mm ∅) containing *ad libitum V. unguiculata* seeds and these assays were placed in an incubator exhibiting the same abiotic conditions described above, thus mimicking the protocol for the inbreeding experiment but in a fashion capable of revealing differences in lifetime offspring production attributable to either female fecundity or male fertility within extreme lineages.

In addition to this follow up experiment, three additional efforts were made to provide further insight regarding extinction patterns across isofemale lines. First, we performed a demographic simulation based solely on estimates of female fecundity from Berger et al. [25]. This simulation provided an expected proportion of lineage extinction per isofemale line attributable to females having little or no fecundity (in which either no offspring, or offspring of only one sex, emerged) at any point during 10 consecutive generations of single-pair matings (see Additional file 1: S3 for details). Second, we calculated the expected proportion of lineage extinction per isofemale line based solely on linear projections of their extinction rates exhibited in the first generation (i.e. upon the establishment of their ~20 replicate lineages). This calculation provides the expected proportion of lineages going extinct for each isofemale line after 10 consecutive generations of single-pair matings without any further inbreeding (see Additional file 1: S4 for details). Lastly, the apparent mode of extinction (e.g. no eggs laid, unhatched eggs, only one sex emerging, etc.) for each lineage going extinct was recorded and later converted to quantitative data that were analyzed in a variety of ways aiming to gain mechanistic insight to patterns of extinction (see Additional file 1: S5 for details). These analyses on the mode of extinction were inconclusive regarding any mechanistic insight to the observed patterns of extinction beyond that reported in the Results section below. Thus, we report and discuss the results of the mode of extinction analyses in Additional file 1: S5.

### Statistical analysis

In order to acquire the derived variables, antagonism and concordance (see Fig. 1), we rotated the coordinate system of the intersexual genetic correlation clockwise 45° as in Berger et al. [25]. This rotation was performed on log-transformed mean fitness for males and females of each isofemale line (collected by [25]), which was variance-standardized (and thus zero-centered) within each sex separately. The resultant antagonism and concordance variables pass through the origin with slopes of −1 and 1 on the original coordinate system, respectively (Fig. 1).

A mixed effects Cox regression was used to analyze variation in extinction risk across isofemale lines (a random effect) in relation to isofemale line values for antagonism and concordance. We also present an alternative mixed effects Cox regression using isofemale line values for female fitness and male fitness (rather than antagonism and concordance) to aid the interpretation of our results. Analyses were performed using the ‘coxme’ package (v.2.2-5, [34]) in R (v.3.2.1, [35]). One extremely male-benefit isofemale line, > 3 SD from the mean for antagonism (Additional file 1: Figure S1a), was a potential outlier in our analyses. There is currently no way, in theory or in practice, to extract residuals or similar from mixed-effects Cox regressions [34], making the assessment of outliers difficult. Thus, the potential outlier was assessed by examining the frequency distribution of differences in fit for the beta coefficients attributable to each datum’s exclusion from a standard Cox proportional hazards model using the ‘survival’ package (v.2.38 [36]) in R [35] (Additional file 1: Figure S1b). Note that the ‘survival’ package’s *cluster* argument does not properly incorporate random effects [34, 36]. Nevertheless, this approach revealed a cluster of observations from the isofemale line in question with a disproportionate influence on the model (Additional file 1: Figure S1b). In the proper mixed effects Cox regressions (from ‘coxme’), removal of this isofemale line provided a superior improvement of models’ fits relative to their respective null models. Whether it was for the antagonism/concordance model or the male-fitness/female-fitness model (since they are the exact same data distributed onto different variables), when the outlier line was included the AIC difference between the fitted and null models was 50.39, whereas without the outlier line the AIC difference was 54.38—the latter being a substantially better fit (i.e. greater reduction in AIC) relative to their respective null models. Thus, this outlier isofemale line was excluded from all analyses (including the follow-up investigations) and derived variables were recalculated in its absence (see Additional file 1: Table S1 and S2 for Cox regressions including this outlier line). Model comparison by log-likelihood ratio tests were used to evaluate whether or not an interaction term should be included in these models (i.e. antagonism*concordance, or male*female). Neither model’s fit was significantly improved by the inclusion of an interaction term (antagonism/concordance: *χ* ^2^ = 0.001, *P* = 0.97; male-/female-fitness: *χ* ^2^ = 0.23, *P* = 0.63).

Female fecundity and male fertility data showed a number of zeros, but were otherwise normally distributed. The likelihood of producing zero eggs did not differ between male-benefit/high-extinction and female-benefit/low-extinction categories in the female fecundity assays (*χ* ^2^ = 0.03, *P* = 0.87) or the male fertility assays (*χ* ^2^ = 0.12, *P* = 0.73). Zero-data were therefore excluded, and general linear mixed models (producing normally distributed residuals) were used to analyze differences in female fecundity (*n* = 153 male-benefit, *n* = 109 female-benefit) and male fertility (*n* = 157 male-benefit, *n* = 126 female-benefit) between individuals descended from the three most female-benefit/low-extinction isofemale lines and the three most male-benefit/high-extinction isofemale lines. Here, lifetime offspring production was the response variable and random effects included isofemale line, lineage nested within isofemale line, and date of the assay. Fixed effects included category (i.e. male-benefit or female-benefit) and base population cohort. These analyses were performed using the ‘lme4’ package (v.1.1-8, [37]) in R [35].

## Results

### Inbreeding experiment

Antagonism was significantly associated with extinction risk (Table 1). Lineages derived from isofemale lines toward the male-benefit/female-detriment end of the spectrum were significantly more likely to go extinct as they became more inbred, and did so more rapidly, relative to lineages derived from isofemale lines toward the female-benefit/male-detriment end of the spectrum (Fig. 2a). Concordance was marginally non-significantly related to extinction risk (Table 1), with a trend toward lineages derived from generally low-fitness (i.e. low-male and low-female fitness) isofemale lines exhibiting greater extinction upon becoming more inbred relative to those derived from generally high-fitness (i.e. high-male and high-female fitness) isofemale lines (Fig. 2b). When instead analyzing sex-specific fitness, female fitness was significantly negatively associated with extinction risk, but there was no significant relationship between male fitness and extinction risk (Table 2).

**Table 1:** Results of a mixed effects Cox regression of extinction risk, with antagonism and concordance as fixed effects, and isofemale line as a random effect

Fixed effects:	Coef.	s.e.	z	*P*
Antagonism	0.20	0.07	2.69	0.007
Concordance	−0.14	0.07	−1.95	0.051
Random effects:	Variance			
Isofemale line	0.14

**Table 2:** Results of a mixed effects Cox regression of extinction risk, with female and male fitness as fixed effects, and isofemale line as a random effect

Fixed effects:	Coef.	s.e.	z	*P*
Female fitness	−0.24	0.08	−3.18	0.002
Male fitness	<0.01	0.08	0.04	0.960
Random effects:	Variance			
Isofemale line	0.14			

### Causes of extinction

There were no significant differences in female fecundity (χ^2^_1_ = 0.55, *P* = 0.46) or male fertility (χ^2^_1_ = 1.50, *P* = 0.22) between female-benefit and male-benefit categories. There were also no differences between categories in the likelihood of producing zero eggs (female fecundity assays: *χ* ^2^ = 0.03, *P* = 0.87; male fertility assays: *χ* ^2^ = 0.12, *P* = 0.73).

Simulations of the expected proportion of extinction across isofemale lines, based solely on the female fecundity of each isofemale line, revealed a pattern of extinction across isofemale lines that was qualitatively similar to that observed (Additional file 1: Figure S2). However, the simulation predicted that only 18.2% of the ~800 lineages would have gone extinct after 10 generations of single pair matings, whereas 77.8% of the lineages actually went extinct. Similarly, calculating the proportion of lineages per isofemale line expected to go extinct based on linear projections of their extinction rates exhibited in the first generation of single-pair (but not full-sib) matings predicted only 19.9% of lineages going extinct (Additional file 1: Figure S2). Both of these efforts to forecast the pattern of extinction that would ensue during this experiment in the *absence* of continued inbreeding leave ~75% of the observed extinction unaccounted for. These efforts show that inbreeding *per se* must have played a major role in generating the observed patterns of extinction across isofemale lines.

## Discussion

The last decade has seen a renewed interest in the effects of sexual selection on population viability, a long-standing issue [38]. While it is currently clear that sexual selection may in theory have both positive [1–6, 39, 40] and negative [22, 41–47] effects, the net effect on a population and whether different effects might interact is much less clear. Our work shows that although strong sexual selection in males may act to purify the genome of SC mutation load, the overall load on population viability may be greater than currently anticipated due to the interaction between the SA gender load and residual SC mutation load, both of which are expected to be left in the wake of strong purifying sexual selection [6, 9, 12]. Specifically, we have shown that male-benefit SA genotypes (isofemale lines) are at greater risk of extinction under inbreeding. Thus, sexual selection may pose two related and interacting costs to populations: (1) the gender load, expected to cause a general depression of population fitness [15, 22–24], and (2) the interaction between gender load and mutation load, for which the present study provides the first empirical insights. There are potentially two reasons for the latter cost being (at least partly) attributable to sexual selection in conventional mating systems [48]: (1) if sexual selection in males does purify some of the SC mutation load and leave behind mostly SA genetic variance for fitness as well as some residual SC mutation load, an evolutionary history of strong sexual selection in males would set the stage for the present findings, and (2) continued sexual selection in males in the context of SA genetic variance for fitness will enrich populations for male-benefit/female-detriment alleles, which, as suggested by the present findings, would render populations more prone to extinction from inbreeding. By this process, sexual selection would tend to prevent the population-level detriments of male-benefit/female-detriment alleles from being canceled out by the population-level *benefits* of female-benefit/male-detriment alleles. However, even if alternative allelic variants at SA loci across the genome were typically in near equal frequencies and their effects on inbreeding depression might cancel out, (small) sub-populations owing to fragmentation or founder effects may still sometimes be enriched for male-benefit/female-detriment allelic variation by chance and therefore suffer enhanced vulnerability to extinction from inbreeding. The mechanistic explanation of how this type of genetic variation may elevate vulnerability to inbreeding remains unclear, but below we discuss some non-mutually exclusive possibilities, and their implications.

### Potential causes of extinction

Female fecundity is a major contributor to population viability in most species with separate sexes [49] and seed beetles are no exception to this rule [24, 50]. At first it may seem that our findings reinforce this notion in that female fitness, but not male fitness, significantly explains variation in extinction from inbreeding (Table 2). However, this difference may be due to the marked environmental variance in male fitness (see Methods). With male and female fitness being highly correlated, our model is likely biased toward finding covariation between extinction and female (rather than male) fitness as it was measured with less error (see Methods and [25]). Nevertheless, one intuitive explanation would be that our results were generated by the demographic consequence of low fecundity exhibited by females from male-benefit/female-detriment genotypes. That is, the risk of lineage extinction should increase with decreasing female fecundity simply due to the risk in low numbers of offspring being all the same sex. However, our simulation of this effect, based solely on fecundity differences across isofemale lines (i.e. without the effects of inbreeding), showed that this would only have generated about one quarter of the extinction we observed. Likewise, a linear continuation of the extinction observed in the first generation of inbreeding (i.e. with very little input from inbreeding yet), would also have generated only about one quarter of the extinction we observed. Even if these two efforts to forecast the expected extinction rates among isofemale lines over 10 consecutive generations of single-pair (but not full-sib) matings represent entirely different mechanisms, then their combined/additive effects would still only have generated about half of the observed extinction, indicating that inbreeding must have played a major role in generating the pattern of extinction we observed. It would of course seem plausible that a risk of extinction posed by the demographic consequences of low fecundity could become exaggerated with increasing homozygosity for SA loci and/or deleterious recessives, and we address this in our explanations below. However, if that were the case, one might expect that extant lineages from the most female-detriment/high-extinction isofemale lines would have lower fecundity than the extant lineages from the most female-*benefit*/low-extinction isofemale lines after 10 consecutive generations of inbreeding, but this prediction was not supported by our follow-up experiment. Note that our follow-up experiment suffered from a complete lack of estimates from some of the most female-detriment/high-extinction isofemale lines, as all 20 of their replicate lineages were extinct after 10 generations of inbreeding, a testament to the difficulty of identifying the proximate causes of extinction [33]. We suggest three additional effects that may have contributed to the increased risk of extinction seen in male-benefit/female-detriment genotypes.

First, our isofemale lines were genetically diverse and male-benefit/female-detriment isofemale lines should, across many loci, harbor higher allele frequencies of male-benefit/female-detriment alleles at SA loci (and the opposite case for female-benefit/male-detriment isofemale lines). This implies that the risk of fixation of male-benefit/female-detriment alleles during inbreeding is higher in male-benefit/female-detriment isofemale lines, and the opposite for female-benefit/male-detriment isofemale lines. With the male-benefit SA isofemale lines of this population already having relatively low female fecundity and population productivity [26], fixation of such alleles via inbreeding would likely further depress female-specific fitness and increase the risk of extinction. We note that this effect would not involve a conventional genetic load maintained by mutation-selection balance [29, 30] but rather a gender load maintained by balancing selection at SA loci. Under this scenario, our analyses (Table 1) would be interpreted as showing independent effects of a SC mutation load, as revealed by the ‘concordance’ dimension, and a SA gender load, as revealed by the ‘antagonism’ dimension, upon the increasing homozygosity that follows from inbreeding. The relative contribution of these different sources of inbreeding depression (mutation load versus loci under balancing selection) has fundamental implications for the classic evolutionary paradox of what maintains genetic variance for fitness [28]. If inbreeding depression is attributable more so to homozygosity for mutation load than to homozygosity for loci under balancing selection, the implication would be that mutation selection balance (a constant influx of mutations and weak selection against them) plays a greater role in maintaining genetic variance for fitness relative to balancing selection at loci of major effect [28]. Oppositely, if inbreeding depression is attributable more so to homozygosity for loci under balancing selection, such loci would be implicated as greater contributors to the maintenance of genetic variance for fitness [28]. Granted our population’s genetic variance for fitness was already known to be dominated by balancing selection at SA loci (recall that this is the theoretical expectation for well-adapted populations at equilibrium, [12, 21]), our analyses would imply that inbreeding depression (and therefore genetic variance for fitness) may to a relatively large extent be attributed to (homozygosity at) loci under balancing selection, a possibility acknowledged early on [51, 52] due the relatively large fitness effects of such loci.

Second, the effective genetic load may differ between the sexes, and this may interact epistatically with SA genetic variation for fitness. Sex differences in the effective genetic load could result from, for example, differences in the number or types of genes that contribute to sex-specific fitness. Immonen et al. [53] recently showed that more than 50% of all genes are differentially expressed in the abdomen of male and female *C. maculatus*, so sex-specificity in the genetic architecture of inbreeding depression is certainly possible. Remarkably, females have previously been shown to be much more affected by inbreeding than males in this species, both in terms of reduced body size [54] and life span [55, 56], congruent with recent findings in *Drosophila* [57]. This suggests that the genetic load across loci encoding for male-specific fitness may be lower, perhaps due to more efficient purging via stronger selection [11, 48] and/or sex-linked mutations being exposed unconditionally in the heterogametic sex [57]. If there is a greater effective load on female-specific function relative to male-specific function, then females with male-benefit SA genotypes (i.e. those starting out with already lower female-specific function) might suffer greater detriments upon inbreeding/homozygosity relative to males with female-benefit genotypes. This sort of epistatic interaction between sex-specific effective mutation load and SA genetic variation could have generated the asymmetric pattern of extinction we observed along the ‘antagonism’ dimension.

Lastly, there could actually be more mutation load linked to male-benefit SA genetic variation than to female-benefit SA genetic variation. Connallon and Jordan [58] have recently shown that, in theory, SA selection should generate female-harming mutations near male-benefit SA alleles, and vice versa. This process would generate a similar mutation load linked to male- and female-benefit SA genetic variation, and would therefore not explain the asymmetric pattern of extinction we observed along the ‘antagonism’ dimension. However, Connallon and Jordan [58] also showed that male-biased mutation rates (a common and widespread phenomenon) should interact with SA selection to generate an increased mutation load on male-benefit haplotypes relative to female-benefit haplotypes. If there is a greater mutation load linked to male-benefit (relative to female-benefit) SA genetic variation in our population, this could explain our observation that male-benefit SA genotypes exhibited greater extinction rates upon inbreeding/homozygosity relative to female-benefit genotypes.

## Conclusions

Independent of the precise causes of extinction, our experiments reveal a previously unappreciated manifestation of the gender load that may result from sexual selection. Whether strong sexual selection in males will tend to increase the relative frequency of SA genotypes in a population depends critically on the extent to which sexual selection favors SC or SA genotypes [6, 12]. Nevertheless, whether due to the purifying capacity of sexual selection [6] or other forms of SC purifying selection, we expect well-adapted populations to exhibit predominantly SA genetic variance for fitness [12, 21], in which case continued sexual selection would favor male-benefit/female-detriment alleles [6, 16]. We have shown here that this male-benefit/female-detriment genetic variation favored by sexual selection in males incites greater inbreeding depression than female-benefit/male-detriment genetic variation. Depending on the underlying mechanism (see above), this means that male-benefit SA genetic architecture, its phenotypic effects, or both pose an additional threat to population viability that has not yet been recognized. Further, in addition to selecting for female-detriment alleles that are sensitive to inbreeding depression, strong sexual selection in males should also decrease effective population size [22, 28, 59]. Thus, under certain conditions, sexual selection may set off an extinction vortex whereby these mechanisms reinforce one another.

Whether strong sexual selection in males poses greater net benefits or detriments to population viability will depend on many interacting factors [6, 59]. Our findings add resolution to the role of SA genetic variation for fitness in determining that outcome. Although we expect natural populations to exhibit mostly SA genetic variance for fitness [12, 19–21], such as the population we used here, we note that other populations of *C. maculatus* show less SA genetic variance [25]. Empirical evidence suggests that natural populations exhibit variable but potentially abundant levels of SA genetic variance for fitness [15, 23, 25, 56, 60–66]. Perhaps owing to these varying levels sexual antagonism, or to the varying methodologies across studies, empirical estimates of the effects of sexual selection on populations show a range of effects, from positive (e.g. [7–11]), to ineffectual (e.g. [67, 68]), and even negative (e.g. [47, 68, 69]; reviewed in [6, 19]). Indeed, Whitlock and Agrawal [6], among others, warn that strong sexual selection in males in the context of SA genetic variance for fitness could have neutral or even negative effects on populations. Our findings add merit to this possibility, showing that in a population exhibiting SA genetic variance for fitness, continued sexual selection for male-benefit/female-detriment genotypes could enhance a population’s risk of extinction under inbreeding depression. This may weaken the general potential for strong sexual selection in males to account for the two-fold cost of sex [1–6].

As a final cautionary note, we have shown here that when constructing inbred lines, a non-random pattern of extinction occurs with regard to sex-specific fitness. Thus, ﻿a panel of inbred lines will exhibit ﻿a non-random representation of segregating genetic variation for fitness. Specifically, SA alleles that are sexually selected in males may be underrepresented or even lacking from panels of inbred lines, such as the *Drosophila* genetic reference panel (DGRP [70]). It remains unclear how far this result can be extrapolated, especially with reference to loci under other forms of balancing selection. Nevertheless, this raises the issue of what types of questions regarding genetic architecture we can expect to validly address using inbred lines. At the very least, our results suggest that we should exercise caution when drawing conclusions based on such panels. One strategy to combat this issue, of course, would be to characterize how populations’ genetic variance for fitness is distributed across genotypes prior to inbreeding them, so as to detect the type of genetic architecture expected to be underrepresented or lacking in a resultant panel of inbred lines.

## Additional files

Additional file 1: Figure S1. Exceptions to sample size per isofemale line—explanation of deviations from a perfectly balanced number of lineages per isofemale line in the inbreeding experiment. **Figure S2.** Special-case extinction criteria—details of the protocol of the inbreeding experiment. **Figure S3.** Simulating expected fecundity-based extinction—details of the follow-up simulation. **Figure S4.** Calculating expected extinction based on first generation—details of the follow-up calculation. **Figure S5.** Modes of extinction—details of the investigation of the varied ways in which lineages went extinct during the inbreeding experiment. (DOC 377 kb)
