# Supplementary material for: Male-benefit sexually antagonistic genotypes show elevated vulnerability to inbreeding
Source: BMC Evol Biol. 2017 Jun 12;17:134. doi: 10.1186/s12862-017-0981-4 (PMC5469140; doi:10.1186/s12862-017-0981-4)
Supplement: Supplementary file 1 — Exceptions to sample size per isofemale line—explanation of deviations from a perfectly balanced number of lineages per isofemale line in the inbreeding experiment. Figure S2. Special-case extinction criteria—details of the protocol of the inbreeding experiment. Figure S3. Simulating expected fecundity-based extinction—details of the follow-up simulation. Figure S4. Calculating expected extinction based on first generation—details of the follow-up calculation. Figure S5. Modes of extinction—details of the investigation of the varied ways in which lineages went extinct during the inbreeding experiment. (DOC 377 kb) [file 12862_2017_981_MOESM1_ESM.doc]

**Additional file 1:**

*S1 Exceptions to sample size per isofemale line:*

By mistake, one isofemale line was only represented by 8 (instead of 20) lineages from the start; the inclusion/exclusion of this isofemale line did not influence any of our analyses. Additionally, 4 observations/lineages (out of ~800) were removed from the analysis because they were somehow mishandled (e.g. a container was mislabeled).

*S2 Special-case extinction criteria:*

The criterion used to determine if a lineage had survived the previous generation was the ability of at least a single virgin full-sib pair from that lineage to copulate. In rare events when adults would/could not mate, pairs were placed on beans without having observed a copulation, and if no eggs were produced by such pairs those lineages were determined to have gone extinct the generation previous to the emergence of those non-mating beetles; if such a pair did produce eggs but no offspring emerged then the lineage was determined to have gone extinct on that generation, and if such a pair did produce offspring then the offspring were treated as usual in terms of determining the lineage’s (generation of) extinction/survival.

*S3 Simulating expected fecundity-based extinction:*

Each isofemale line had ~12 estimates of female fitness/fecundity from Berger *et al.* [25], some of which were quite low (or even zero), low enough to cause a lineage to go extinct if by chance all offspring produced were of the same sex. We used these data to generate poisson distributions of each line’s fecundity, from which 10,000 estimates of fecundity were drawn for each line. The value of each of these estimates (representing the number of offspring produced) determined the number of times to draw from a binomial distribution (representing the probability that each offspring was either male or female). For each isofemale line, the proportion of events resulting in either single-sex offspring or zero offspring represents the expected proportion of single-pair matings for a given line that would result in extinction due to few or no offspring. This proportion was raised to the power of 10 to represent the 10 consecutive generations of single-pair matings for which this potential cause of extinction could have ensued. The resultant value for each isofemale line represents the expected proportion of lineage extinctions per isofemale line attributable to little or no offspring being produce at any point during 10 consecutive generations of single pair matings (without increasing homozygosity). These proportions were compared to the actual proportion of lineages that went extinct for each line (see Results and Figure S2). This simulation was performed in R (v.3.0.1, [35]).

*S4 Calculating expected extinction based on first generation:*

As an additional means of calculating the expected/null pattern of extinction, we took the proportion of lineages per isofemale line that had gone extinct after the first generation of single-pair matings (i.e. during the establishment of the 20 lineages for each isofemale line), which were not full-sib matings but exhibited the low level of inbreeding present in isofemale lines—that proportion was raised to the power of 10. The resultant value provides an estimate of the proportion of lineages from each isofemale line that would have gone extinct after 10 generations according to a linear continuation of the extinction exhibited by each line in the first generation (i.e. if the inbreeding coefficient would have remained constant at the low level exhibited by the isofemale lines). This was compared to the actual proportion of lineages from each isofemale line that went extinct after 10 generations of inbreeding (see Results and Figure S2).

*S5 Modes of extinction:*

(a) Methods

The way in which each lineage extinction occurred was recorded anecdotally throughout the inbreeding experiment and later quantified according to a hierarchical scale ranging from 1 to 8: (1) no eggs, (2) < 6 eggs, (3) 6-12 eggs, (4) > 12 eggs, (5) windows on seeds (indicating a developing beetle inside), (6) deformed (or sexually ambiguous) adults, (7) adults of only one sex, and (8) adults of both sexes (which ultimately couldn’t/wouldn’t mate). These data were analyzed in a variety of ways in search of processes underlying patterns of extinction. Below we highlight the only notable results from these analyses, and discuss/interpret those results.

(b) Results and Discussion

A multiple regression of the antagonism and concordance variables on the number of different modes of extinction for each isofemale line revealed that lineage extinctions from female-benefit genotypes happened in a greater variety of ways relative to male-benefit genotypes (*F*_1,37_ = 8.6, *P* = 0.006; Figure S3), and generally high-fitness genotypes went extinct by a greater variety of ways relative to generally low-fitness genotypes (*F*_1,37_ = 4.2, *P* = 0.047). The same multiple regression, but using male and female fitness instead of antagonism and concordance, revealed that high female-fitness genotypes went extinct by a greater variety of ways relative to low female-fitness genotypes (*F*_1,37_ = 11.5, *P* = 0.001), but there was no relationship for male fitness (*F*_1,37_ = 0.01, *P* = 0.935). The most common mode by which lineages went extinct was that there were very few or no eggs (i.e. mode 1, Figure S3); the second most common mode of extinction was that adults of only one sex or the other emerged (i.e. mode 7, Figure S3). This second most common mode of extinction (in which only one sex emerges, hereafter: sex-biased extinction) is obviously related to the first, since instances of very few eggs are prone to all offspring being of one sex or the other by random chance. This process was integral to the simulation based on female fecundity (see S3) in order to predict the likelihood of sex-biased extinctions attributable to low fecundity. That simulation predicted a negative relationship between antagonism and the proportion of lineages per isofemale line surviving 10 generations of single-pair matings (see Results and Figure S2). However, multiple regressions on the observed data revealed no significant effects of antagonism, concordance, male fitness, or female fitness on the (square root transformed) frequency or (arcsine square root transformed) proportion of isofemale lines’ extinction events characterized by sex-biased extinction (all *F*s_1,35_ < 0.44, all *P*s > 0.5). For those isofemale lines exhibiting sex-biased lineage extinctions (n=38), multiple regressions revealed no effect of antagonism, concordance, male fitness, or female fitness on mean sex ratio (i.e. the direction and magnitude of skew toward one sex or the other emerging; all *F*s_1,35_ < 0.13, all *P*s > 0.7). Analyzing the absolute value of each isofemale line’s mean sex ratio of their sex-biased extinctions (i.e. the magnitude but not direction of skew toward one sex or the other emerging) revealed that this skew was significantly greater in female-benefit isofemale lines than in male-benefit isofemale lines (*F*_1,35_ = 6.4, *P* = 0.016; Figure S4a), but was not related to concordance (*F*_1,35_ = 0.1, *P* = 0.762). The same multiple regression, but using male and female fitness instead of antagonism and concordance, revealed low male-fitness genotypes to have a non-significantly greater absolute value sex ratio than high male-fitness genotypes (*F*_1,35_ = 3.01, *P* = 0.091) and no relationship regarding female fitness (*F*_1,35_ = 1.5, *P* = 0.222). However, when removing those extinction events in which only one offspring emerges, which can *only* be of one sex or the other (reducing the sample size to n=29 isofemale lines), all covariates have a non-significant relationship to mean absolute value sex ratio (log transformed mean sex ratio: all *F*s_1,26_ < 1.09, all *P*s > 0.3; Figure S4b). These analyses were performed using JMP (v. 10.0.0; SAS Institute Inc., 2012).

Lineages from female-benefit and generally high-fitness genotypes went extinct in a greater variety of ways (e.g. no eggs, unhatched eggs, no adults, adults of only one sex). This may indicate that female-benefit and “good-quality” genetic architecture was allowing beetles (of both sexes) to make if further through the developmental cascade (i.e. those genotypes were better equipped to buffer the stress of development) as lineages became more inbred. Alternatively, one could interpret this as those genotypes causing lineages to fail at a greater variety of developmental stages. So this cannot yield any conclusive insights.

Extinction events in which only one sex or the other emerged tended to exhibit greater (absolute value) deviations from a 1:1 sex ratio when exhibited by lineages from female-benefit genotypes than those from male-benefit genotypes. This effect should be interpreted with caution since it did disappear when excluding those observations in which only one offspring emerged (i.e. those extinctions constrained to producing one sex or the other). This result does not lend any obvious insights. First, it was lineages from female-benefit genotypes (those with greater survival) that exhibited this relatively greater skew in sex ratio upon sex-biased extinctions; and second, it was the absolute value of the skew that was significant—both sexes were equally probable to emerge as offspring. A less obvious explanation for how the sex ratio skew in female-benefit genotypes might support particular scenarios would be that females from male-benefit genotypes were typically doing so poorly that extinction events from male-benefit genotypes were characterized by there being no eggs, whereas females from female-benefit genotypes were, upon going extinct, at least able to lay very few eggs, generating extinction events characterized by few offspring, which, by chance, were sometimes all the same sex. Even these rather speculative interpretations provide no real insight to the mechanism(s) underlying patterns of extinction across isofemale lines.

*Tables:*

| Table S1: Results of the antagonism/concordance mixed effects Cox regression including the outlier isofemale line. | | | | | |
| --- | --- | --- | --- | --- | --- |
|  |  | | | |  |
| Fixed effects: | Coef. | s.e. | z | *P* |  |
| Antagonism | 0.14 | 0.08 | 1.78 | 0.075 |  |
| Concordance | -0.12 | 0.08 | -1.59 | 0.11 |  |
|  |  |  |  |  |  |
| Random effects: | Variance |  |  |  |  |
| Isofemale line | 0.17 |  |  |  |  |
|  | |  |  |  |  |

| Table S2: Results of the male-/female-fitness mixed effects Cox regression including the outlier isofemale line . | | | | | |
| --- | --- | --- | --- | --- | --- |
|  |  | | | |  |
| Fixed effects: | Coef. | s.e. | z | *P* |  |
| **Female fitness** | **-0.19** | **0.08** | **-2.3** | **0.021** |  |
| Male fitness | -0.03 | 0.08 | -0.38 | 0.7 |  |
|  |  |  |  |  |  |
| Random effects: | Variance |  |  |  |  |
| Isofemale line | 0.17 |  |  |  |  |
|  | |  |  |  |  |

*Figures:*

Figure S1:


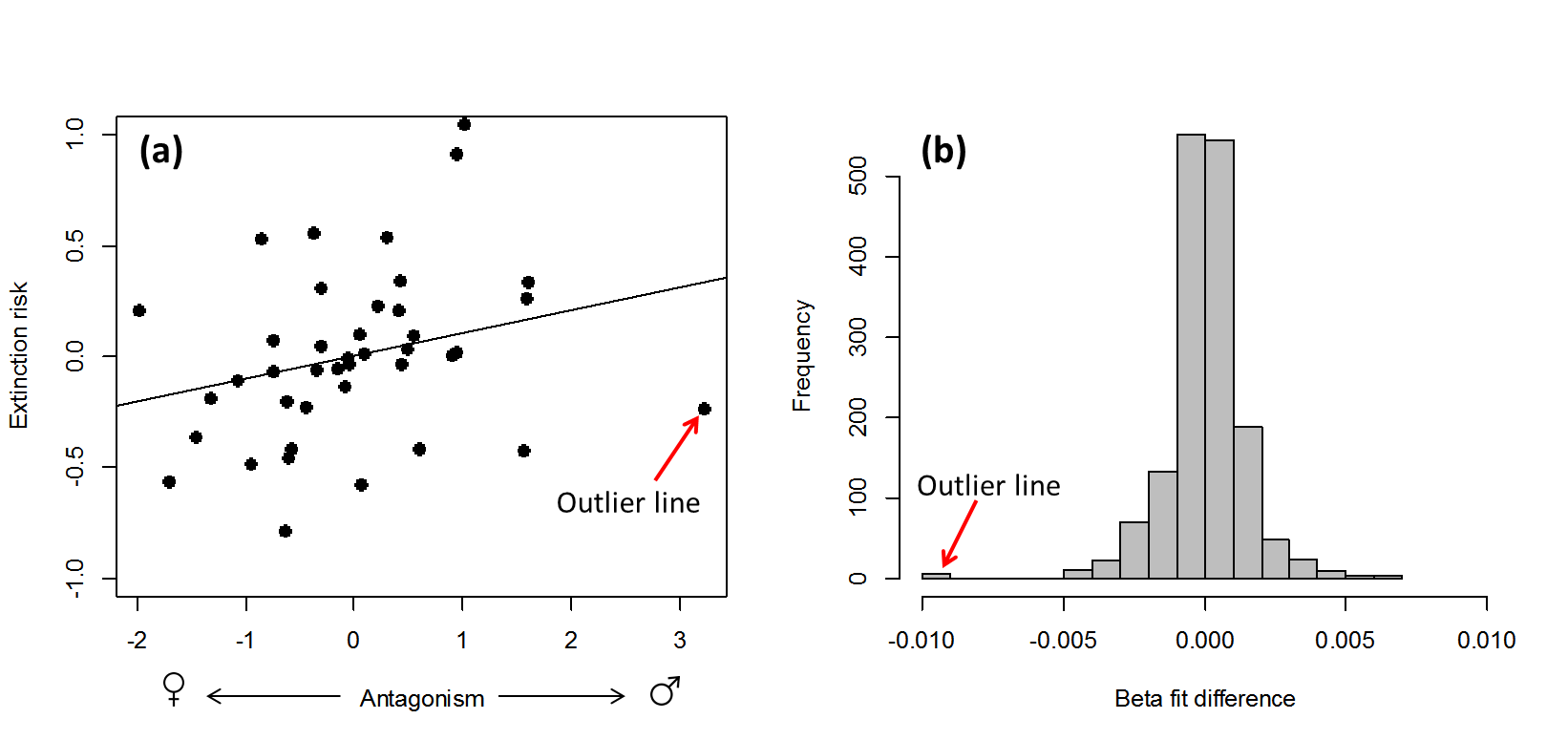


Figure S2:


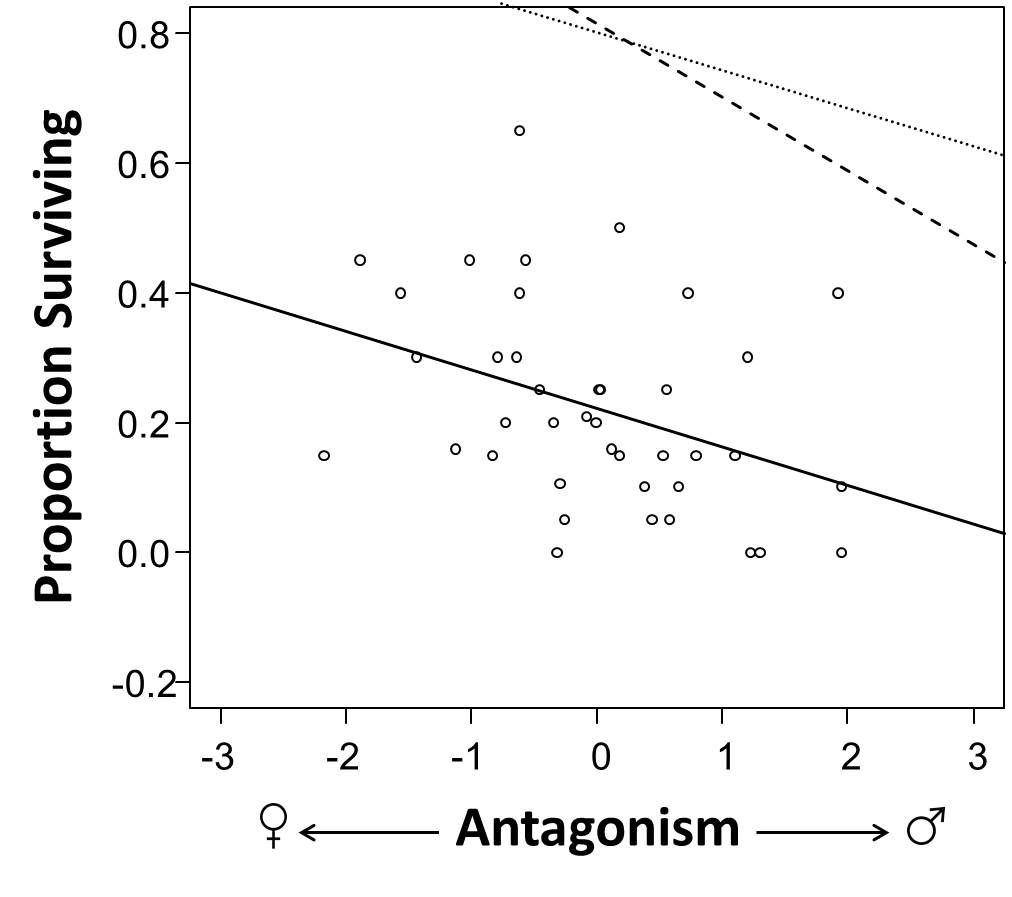


Figure S3


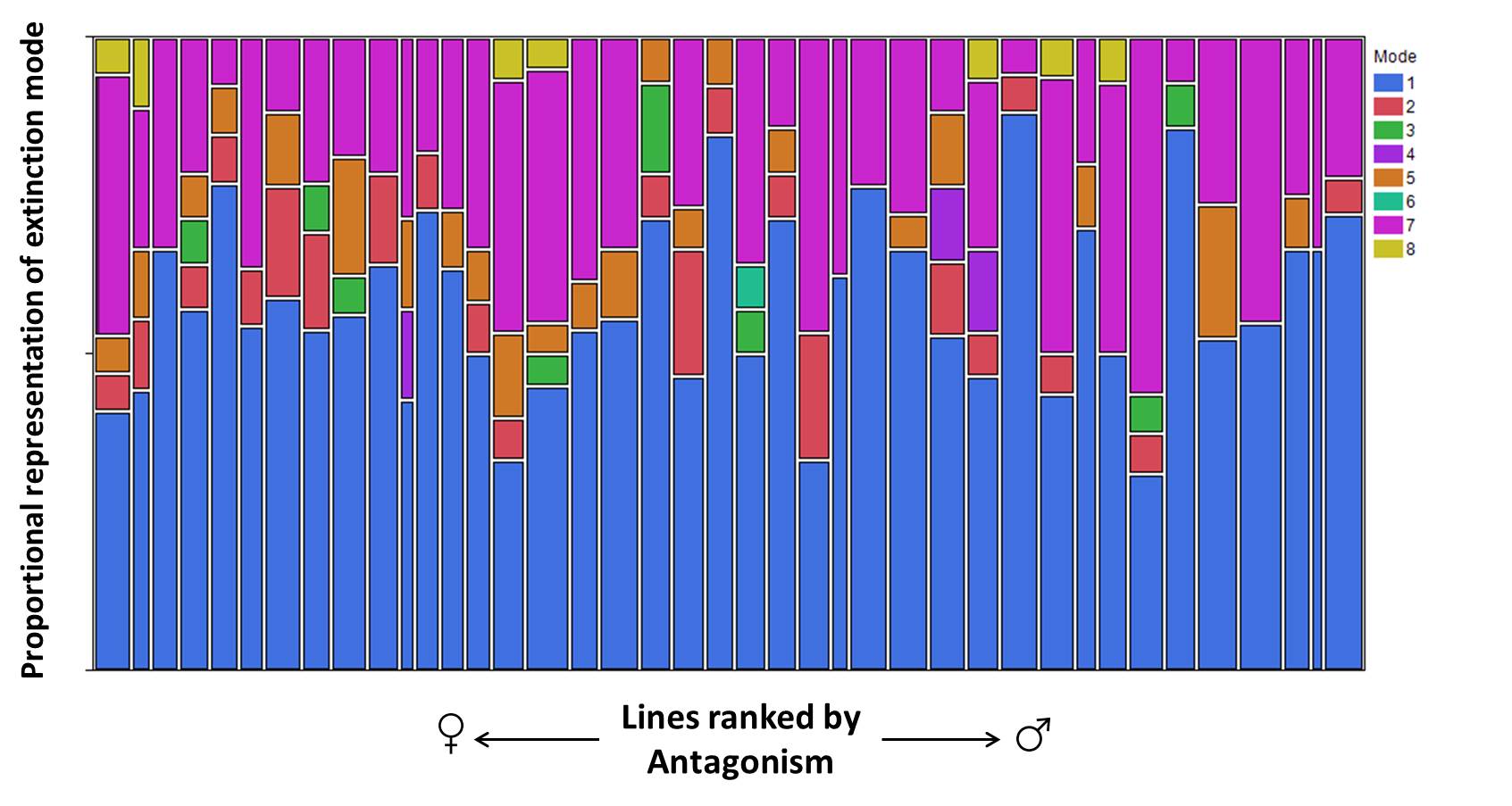


Figure S4


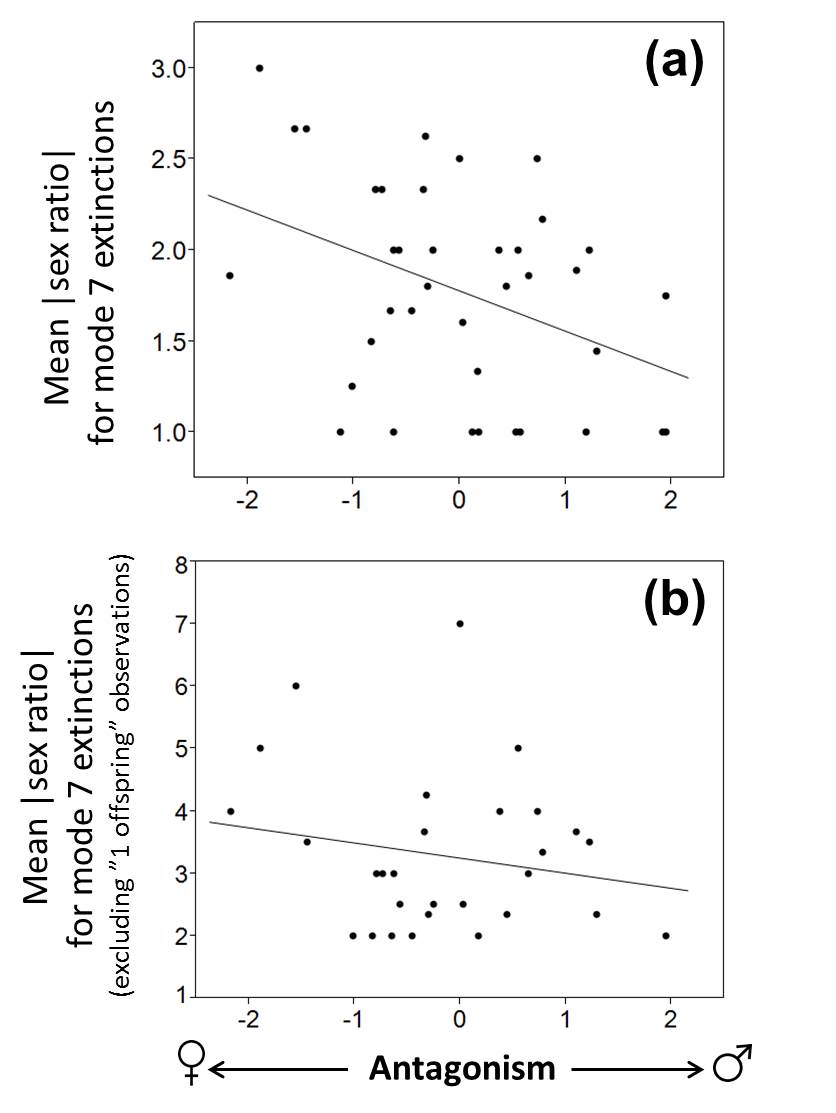


*Figure captions:*

Figure S1: Visual representation of data from the outlier isofemale line (red arrows), shown in (a) a scatter plot equal to Figure 1a but which includes the outlier positioned > 3 SD away from the mean for Antagonism, and (b) a frequency histogram of the differences in fit for the beta coefficients attributable to each datum’s exclusion from a standard Cox proportional hazards model (see Methods).

Figure S2: Negative relationship between Antagonism and the proportion of lineages per isofemale line that survived 10 consecutive generations of single-pair full-sib inbreeding (solid line and plotted data points). The simulated proportion of lineages per isofemale line surviving 10 generations of single-pair matings (dashed line) and the calculated proportion of lineages from each isofemale line surviving 10 generations of single-pair matings (dotted line) predict qualitatively similar patterns of extinction, but both predict only ~20% of lineages going extinct after 10 generations, whereas ~80% of lineages actually went extinct (leaving ~75% of the observed extinction unaccounted for)—showing that inbreeding *per se* did indeed play a major role in generating the observed pattern of extinction among isofemale lines.

Figure S3: Summary figure of the modes of extinction exhibited by lineages from each isofemale line. Isofemale lines are represented by vertical columns, arranged on the x axis according to their rank order along the axis of Antagonism. The widths of the columns are proportional to the number of lineage extinctions exhibited by each isofemale line—isofemale lines to right, being relatively more male-benefit, have thicker columns, representative of our main finding that male-benefit isofemale lines exhibited greater extinction. The y axis is the proportional representation of the different modes of extinction (see S5). Most extinctions occurred due to few or no eggs (mode 1), followed by sex-biased extinctions (mode 7). Female-benefit isofemale lines went extinct by a greater variety of modes relative to male-benefit lineages (S5).

Figure S4: Mean absolute-value sex ratio for mode 7 extinction events (i.e. sex-biased extinction; see S5) for each isofemale line that exhibited such extinctions, plotted against the axis of antagonism. Sex-biased extinctions of female-benefit isofemale lines exhibited significantly greater mean deviations from 1:1 sex ratios (but not consistently biased toward one sex or the other; see S5) relative to sex-biased extinctions of male-benefit isofemale lines (a), but this effect was not significant when excluding extinction events characterized by only one offspring emerging (i.e. those cases constrained to producing one sex or the other) (b).
